# Noma disease: 10 years of research in the quest of a microbial etiology

**DOI:** 10.1186/2047-2994-4-S1-P258

**Published:** 2015-06-16

**Authors:** P François, K Whiteson, D Baratti Mayer, J Schrenzel, D Pittet, the GESNOMA study group

**Affiliations:** 1 Medical Specialties, Univ Hosp of Geneva, Geneva, Switzerland; 2 Mol Biol & Biochem, Unive of California, Irvine, USA; 3 Plastic Surgery, Univ Hosp of Geneva, Geneva, Switzerland; 4 Dep. Medical Specialties, Univ Hosp of Geneva, Geneva, Switzerland; 5 Cont & Prev Infection, Univ Hosp of Geneva, Geneva, Switzerland

## Introduction

Noma is a devastating ancient illness that causes severe facial disfigurement in >140,000 children every year mainly in Africa, South America and India. The cause of noma remains unknown but infection, oral hygiene and immune status likely all contribute

## Objectives

Efforts have been deployed over the 2 past decades to identify microbial agents (fungal, bacterial and viral) responsible for noma

## Methods

Research in the field of microbial identification has benefited from dramatic technical improvements. Until the late 90’s culture based methods were predominantly used. More recently, culture-independent methods were used to identify bacteria in noma patients. We present three approaches used to study hundreds of gingival samples from noma patients and local control samples collected from villages near Zinder, Niger (Africa): large-scale cloning sequencing methods used at the beginning of 2000, high-density microarrays and high-throughput sequencing (HTS) devices

## Results

Culture-based methods identified *Fusobacterium necrophorum* as the causative agent of noma, while culture-independent methods identify higher levels of Fusobacteriales in healthy controls. Cloning-sequencing strategies identify disequilibrium in microbial communities from noma patients particularly in Fusobacteria, *Prevotella intermedia* and *Peptostreptococcus* genus abundance. This was also detected in studies using semi-quantitative microarrays. More recently, the utilization of HTS provided a detailed analysis of microbial flora in noma patients and identified Clostridiales, Bacteroidales, and Spirochaetales as indicators of noma. Our epidemiological investigations excluded the possible role of viruses such as cytomegalovirus or morbillivirus as possible significant contributor of noma disease

## Conclusion

Although we identify a unique distribution and composition of microbial communities in noma wound sites compared to unaffected samples from the same mouths and healthy controls, the etiology of noma disease remains elusive. Future studies should include longitudinal sampling in high risk areas and detailed exploration of microbiota before and during development of lesions

## Disclosure of interest

None declared.

